# Prevalence of diverse colorectal polyps and risk factors for colorectal carcinoma in situ and neoplastic polyps

**DOI:** 10.1186/s12967-024-05111-z

**Published:** 2024-04-17

**Authors:** Xiaojuan Li, Mengting Hu, Zhangjun Wang, Mei Liu, Ying Chen

**Affiliations:** 1 Department of Gastroenterology, Minhang Hospital, Fudan University, Shanghai, 201199 China; 2 Department of General Medicine, Minhang Hospital, Fudan University, Shanghai, 201199 China; 3 Proprietary Trading Department, Huaan Securities Co., Ltd, Shanghai, 200120 China

**Keywords:** Prevalence, Colorectal polyps, Neoplastic polyps, Colorectal carcinoma in situ, Tumor markers

## Abstract

**Background:**

Most colorectal cancers originate from precancerous polyps. This study aimed to determine the prevalence of colorectal polyps with diverse pathological morphologies and to explore the risk factors for colorectal carcinoma in situ (CCS) and neoplastic polyps.

**Methods:**

Inpatients admitted from January 2018 to May 2023 were screened through the hospital information system. Polyps were classified according to pathological morphology. The prevalence of polyps was described by frequency and 95% confidence interval. Univariate and multivariate logistic regression analyses were used to explore the risk factors for CCS and neoplastic polyps.

**Results:**

In total, 2329 individuals with 3550 polyps were recruited. Among all patients, 76.99% had neoplastic polyps and 44.31% had advanced adenomas. Tubular adenoma had the highest prevalence at 60.15%, and the prevalence of CCS was 3.86%. Patients with a colorectal polyp diameter ≥ 1.0 cm or number ≥ 3 were 8.07 times or 1.98 times more likely to develop CCS than were those with a diameter < 1.0 cm or number < 3, respectively (OR 8.07, 95%CI 4.48–14.55, *p* < 0.0001; and OR 1.98, 95%CI 1.27–3.09, *p* = 0.002). The risk of CCS with schistosome egg deposition was also significantly increased (OR 2.70, 95%CI 1.05–6.98). The higher the levels of carbohydrate antigen (CA) 724 (OR 1.01, 95%CI 1.00–1.02) and CA211 (OR 1.16, 95%CI 1.03–1.32) in patients with colorectal polyps were, the greater the risk of CCS. When colorectal neoplastic polyps were analyzed, we discovered that for each 1-year increase in age, the risk of neoplastic polyps increased by 3% (OR 1.03, 95%CI 1.02–1.04), *p* < 0.0001. Patients with a polyp diameter ≥ 1.0 cm had a 2.11-fold greater risk of neoplastic polyps compared to diameter < 1.0 cm patients (OR 3.11, 95%CI 2.48–3.92), *p* < 0.0001. In addition, multiple polyps and CA199 levels are risk factors for neoplastic polyps.

**Conclusion:**

More than 3/4 of colorectal polyp patients have neoplastic polyps. Patients are more inclined to develop CCS and neoplastic polyps if they have large polyps (> 1.0 cm) or multifocal polyps. The levels of the tumor markers CA724 and CA211 show some potential usefulness for predicting CCS and may be exploited for early identification of high-risk populations.

## Introduction

Cancer morbidity and mortality are growing rapidly worldwide. Colorectal cancer accounts for 10.0% of all cancers worldwide. Its mortality rate accounts for 9.4% of total cancer deaths, making it the second leading cause of cancer death [1]. Research estimated that there were approximately 4292,000 newly diagnosed cancer cases and 2814,000 cancer deaths in China in 2015, which equates to an average of approximately 12,000 newly diagnosed cancer cases and more than 7,500 cancer deaths every day. Colorectal cancer is one of the primary causes of new cases and deaths from cancer, placing significant strain on the public health system [2].

The pathways associated with colorectal cancer include chromosome instability, microsatellite instability and serrated neoplasia pathways [3]. Colorectal cancers can be divided into familial and sporadic tumors, with more than 90% of colorectal cancers being sporadic and familial adenomatous polyposis accounting for less than 1%. Most cases involve the malignant transformation of benign adenomas, known as the adenoma-carcinoma sequence. An adenoma diameter ≥ 1 cm is a risk factor for carcinogenesis [4].

Colorectal polyps are protrusions visible on the surface of normal colorectal mucosa and are the precursors of most colorectal cancers. Some colorectal polyps accumulate enough mutations, develop severe dysplasia, and eventually infiltrate the submucosa to develop colorectal cancer [5]. Histologically, these tumors are divided into neoplastic and nonneoplastic polyps. Neoplastic polyps, namely, adenomas, are divided into benign and malignant polyps (adenoma containing aggressive cancer). Adenomatous polyps have malignant potential, are common in adults, and are mostly benign at the time of detection. Adenomas have a 3–5% chance of developing into cancer, whereas nonneoplastic polyps do not have malignant potential [6, 7].

A Swedish study analyzed 178,377 patients with colorectal polyps with a median follow–up of 6.6 years. At 10 years of follow-up, the cumulative incidence of colorectal cancer was 1.6% in patients with hyperplastic polyps, 2.5% in patients with sessile serrate polyps, 2.7% in patients with tubular adenoma, 5.1% in patients with tubulovillous adenoma and 8.6% in patients with villous adenoma, while the incidence of colorectal cancer in the general population of the control group was 2.1%. At 10 years, the mortality of colorectal cancer in the control group was 0.7%, that of hyperplastic polyps was 0.4%, that of sessile serrate polyps was 1.0%, that of tubulovillous adenoma was 1.6%, and that of villous adenoma was 3.5% [8]. The study showed that the incidence of colorectal cancer in the polyp group was greater than that in the control group except for hyperplastic polyps (*p* < 0.0001). However, only tubulovillous adenoma and villous adenoma patients had higher mortality. In addition, the prevalence of sessile serrated polyps in different regions is 2.6–10.5%, and its prevalence increases with age, relatively, + 1.9% per year of age [9].

Although colonoscopy has been widely used as an early screening method for colorectal cancer, it cannot be used to evaluate the factors that cause the occurrence and development of colorectal polyps or cancers. Recurrence after resection of colorectal polyps is also common, with a recurrence risk of 20–50% [10]. The progression from adenoma to colorectal cancer is a lengthy, multistep process involving the accumulation of driver mutations. The detection and resection of colorectal polyps, this process is effective at reducing the overall risk of colorectal cancer [7]. By analyzing the relevant factors of patients with high-risk polyps, high-risk patients can be adequately monitored, and over-monitoring of low-risk patients can be avoided.

The current research tends to study the mechanism of colorectal polyps in colorectal cancer and the relevant factors of neoplastic polyps. Research on the prevalence of various pathological forms of polyps, the significant determinants of very early tumors, and the alteration of blood biomarkers is still lacking. To better comprehend the prevalence of colorectal polyps, risk factors for colorectal carcinoma in situ (CCS) and neoplastic polyps should be identified earlier, and early intervention should be applied in the development and occurrence of colorectal cancer. The purpose of this study was to first assess the prevalence of colorectal polyps with diverse pathological morphologies, then investigate the effects of demographic data, metabolic disease and hematological indicators on CCS and neoplastic polyps, and finally identify the risk factors for the development of CCS and neoplastic polyps.

## Methods

### Study design

This was a retrospective cross-sectional study. Patients were identified from January 2018 to May 2023, through the use of the hospital information system of Minhang Hospital, Fudan University. The screening keywords used were “colonoscopy”, “colon polyps”, “rectal polyps”, “multiple colon/rectal polyps”, “benign colon/rectal tumors”, “colon/rectal carcinoma in situ” and “intestinal carcinoma in situ”. The inclusion criteria were as follows: (1) ≥18 years of age; (2) colonoscopy detect colorectal polyps and endoscopic treatment; and (3) complete demographic and pathological data and colonoscopy records. Exclusion criteria include: (1) malignant polyps or colorectal cancer that requires surgical intervention; (2) complicated with other systemic malignant tumors or acute diseases; (3) intestinal inflammatory disease or infectious colitis; (4) familial polyposis syndrome; (5) missing clinical data.

### Patient selection procedure

**Fig. 1:**
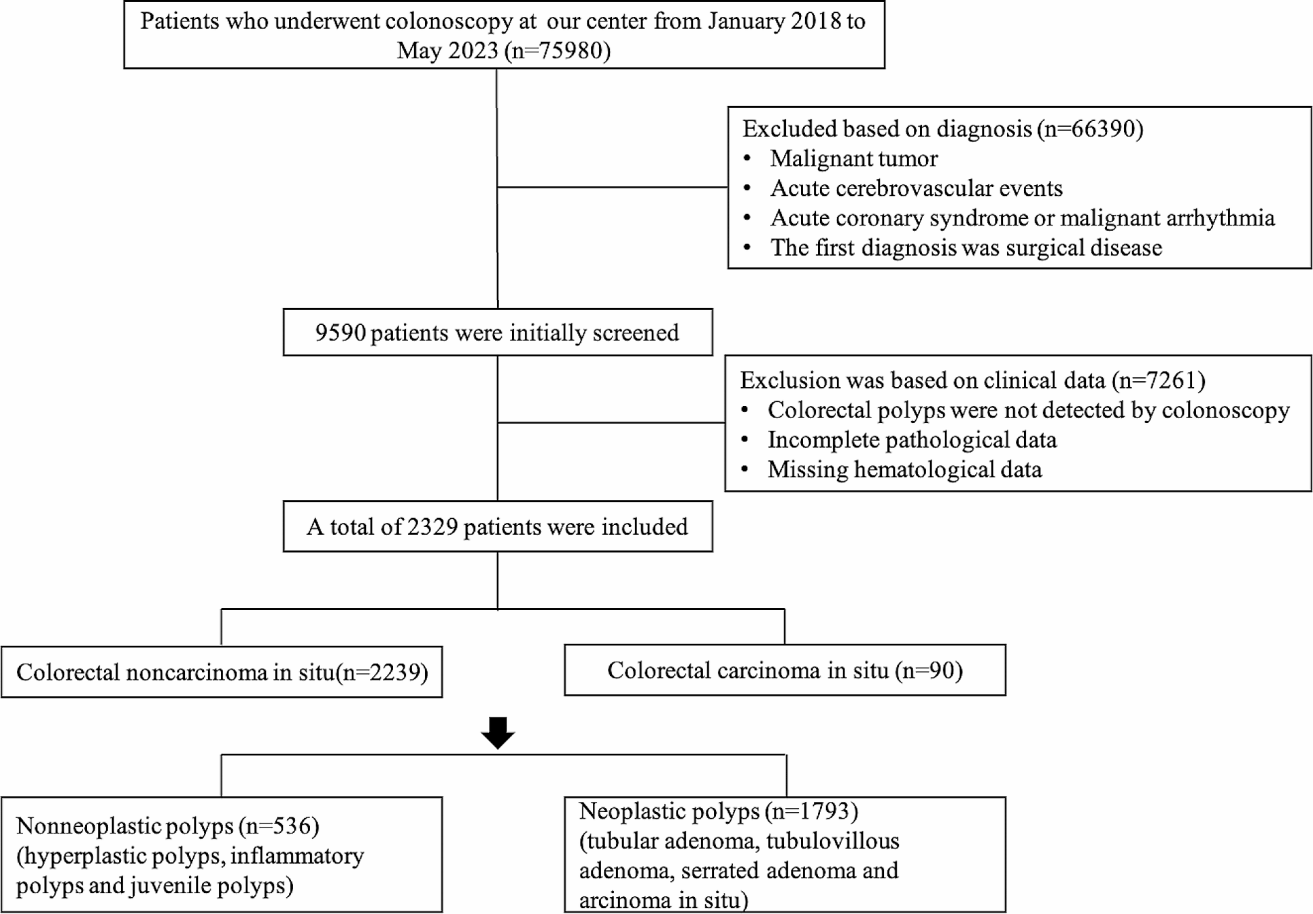
Flow chart of screening patients with colorectal polyps

A total of 75,980 patients were screened through the hospital information system (Fig. 1). 95,950 patients were initially screened according to disease diagnosis. Among them, 5798 patients who underwent colonoscopy had no evidence of colorectal polyps or insufficient pathological data, and 1463 patients with incomplete hematological data were excluded. Ultimately, 2329 patients were included in the study. There were 2239 patients with colorectal noncarcinoma in situ polyps and 90 patients with CCS. Among all patients with colorectal polyps, 1793 had neoplastic polyps, and 536 had nonneoplastic polyps.

### Classification of colorectal polyps

According to the number of polyps detected by colonoscopy, patients were divided into solitary polyps (only one polyp), multiple polyps (≥ 2 polyps), and polyps < 3 or ≥ 3. The pathological diagnosis was extracted from the pathological database records and referenced to the 2010 World Health Organization (WHO) Classification of Tumors of the Digestive System [11]. Histopathologically, polyps were classified as hyperplastic polyps, inflammatory polyps, juvenile polyps, tubular adenoma, tubulovillous adenoma, serrated adenoma, and CCS (some adenomas with high-grade intraepithelial neoplasia or severe dysplasia without breaking through the mucosa lesions confined to the mucosa). According to the histological classification, nonneoplastic polyps include hyperplastic polyps, inflammatory polyps, and juvenile polyps. Neoplastic polyps are divided into adenomas and carcinomas [12]. Traditional adenomas (including tubular adenoma, tubulovillous adenoma and villous adenoma, with or without high-grade intraepithelial neoplasia or severe dysplasia) can be further divided into advanced (≥ 10 mm, severe dysplasia, with tubular villous or villous histology) and nonadvanced adenomas [13]. We calculated all polyp pathologic diagnoses of multiple polyps, whichever occurred with the largest diameter polyp when analyzing the population.

### Data collection

Based on the hospital information system, we collected demographic information, including sex, age, metabolic-associated fatty liver disease (MAFLD), hypertension, diabetes, coronary heart disease, *Helicobacter pylori* infection, and schistosome egg deposition (the schistosomiasis egg deposits were extracted only from pathological diagnosis, and we did not distinguish the species of schistosomiasis). The polyp information included number, diameter and pathology. Hematological data included red blood cell, hemoglobin, white blood cell, platelet, creatinine, uric acid, total bilirubin, aspartate aminotransferase, alanine aminotransferase, albumin, γ-glutamyl transpeptidase, lactate dehydrogenase, alkaline phosphatase, triglycerides, total cholesterol, carcinoembryonic antigen (CEA), alpha fetoprotein (AFP), carbohydrate antigen (CA) 125, CA199, CA724 and CA211.

### Statistical analysis

Frequencies are used to describe categorical variables. We computed 95% confidence intervals (CIs) using the frequency of occurrence of diverse polyps separately. The chi-square test or Fisher’s exact test was used for comparisons between noncarcinoma in situ and CCS, or nonneoplastic polyp and neoplastic polyp. The Kolmogorov-Smirnov test was used to determine whether continuous variables had a normal distribution before describing the data as the mean ± standard deviation or the median and interquartile range (M[IQR]), and the t test or Mann‒Whitney U test was used to compare the two groups. To further investigate the variables that may affect colorectal carcinoma in situ and neoplastic polyps, the variables with statistically significant differences were added to the logistic regression analysis. The goodness of fit was assessed using the Hosmer and Lemeshow test. A difference was considered to be statistically significant at a two-sided *p* < 0.05. The data were analyzed using SPSS (version 20.0), while the graphics were created using GraphPad Prism and R software.

## Results

### Participants’ clinical features and the prevalence of diverse colorectal polyps

This cross-sectional study included 2329 patients. Only counted once for each pathological type was counted for patients with multiple polyps, totaling 3550 polyps. There were 7 pathological types, including hyperplastic polyps, inflammatory polyps, juvenile polyps, tubular adenoma, tubulovillous adenoma, serrated adenoma and CCS. In this study, 64.92% of the patients were male (1512/2329), with a mean age of 60.38 ± 12.08 years. A total of 1389 patients (59.64%, 95% CI 57.65–61.63%) and 1044 patients (44.83%, 95% CI 42.80–46.85%) were older than 60 and 65 years old, respectively. 59.21% (95% CI 57.21–61.21%) of colorectal polyps were between 0.5 and 1.0 cm in diameter (1379/2329), and 59.42% (95% CI 57.43–61.42%) were < 1 cm in diameter (1384/2329), 11.72% (95% CI 10.41–13.03%) were < 0.5 cm in diameter (273/2329), and only 12.54% (95%CI 11.19–13.88%) were > 2.0 cm in diameter (292/2329).

**Fig. 2:**
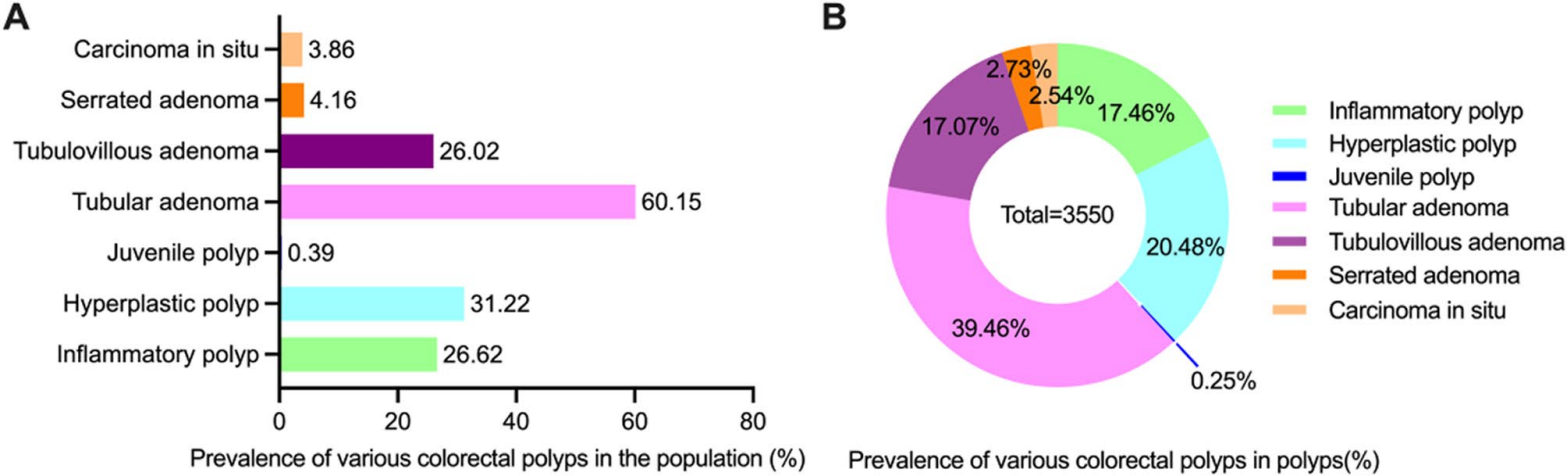
Prevalence of colorectal polyps with diverse pathological morphology

Among all patients with colorectal polyps, 1196 had solitary polyps, a prevalence of 51.35% (95% CI 49.32–53.38%), and 1133 had multiple polyps, a prevalence of 48.65% (95% CI 46.62–50.68%). In the population, the prevalence of diverse pathological type polyps were 60.15% (95%CI 58.16–62.14%) for tubular adenoma(1401/2329), 31.22% (95%CI 29.33–33.10%) for hyperplastic polyps(727/2329), 26.62% (95%CI 24.82–28.42%) for inflammatory polyps(620/2329), 26.02% (95%CI 24.24–27.80%) for tubulovillous adenoma (606/2329), 4.16% (95%CI 3.35–4.98%) for serrated adenoma(97/2329), 3.86% (95%CI 3.08–4.65%) for CCS (90/2329), and 0.39% (95%CI 0.13–0.64%) for juvenile polyps(9/2329) (Fig. 2A). Among the 3550 polyps, tubular adenoma had the highest prevalence (39.46%, 95% CI 37.86–41.07%; 1401/3550), whereas juvenile polyps had the lowest prevalence (0.25%, 95% CI 0.09–0.42%). The prevalence of CCS was 2.54% (95% CI 2.02–3.05%) (90/3550), and the prevalence of other pathological types of polyps is shown in Fig. 2B.

### Prevalence of neoplastic polyps and advanced adenomas in all age groups

**Fig. 3:**
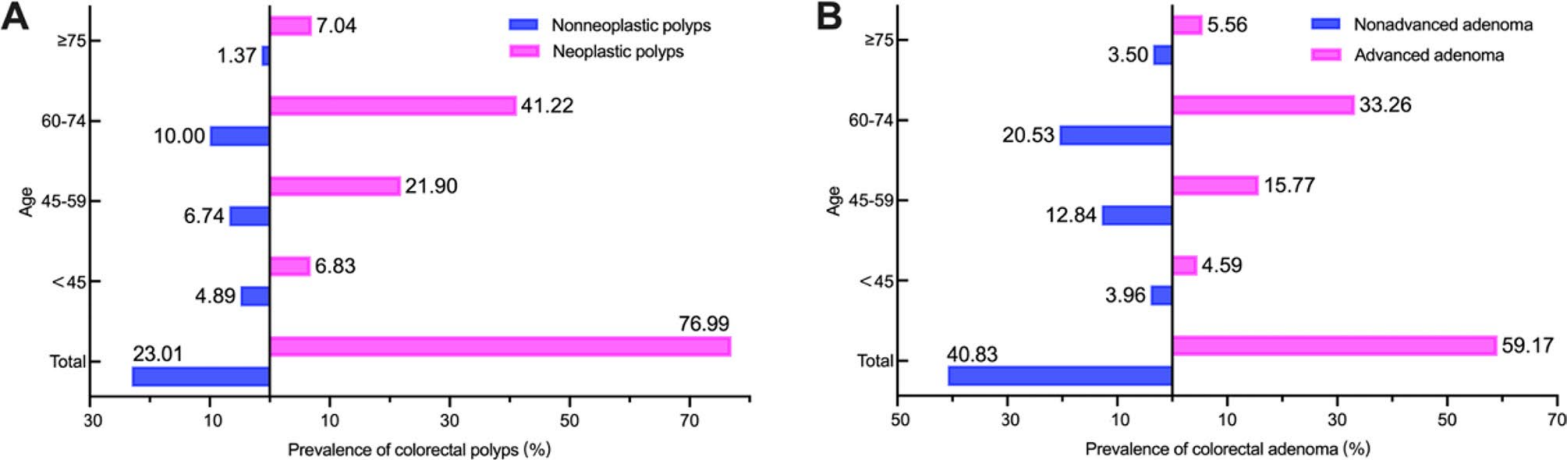
Prevalence of colorectal polyps with diverse pathological morphology in different age groups. **A**: Neoplastic polyps and nonneoplastic polyps. **B**: Advanced and nonadvanced adenomas

Age was divided into < 45 years 45–59 years, 60–74 years, and ≥ 75 years according to the WHO criteria. Among the 2329 patients with colorectal polyps, nonneoplastic polyps accounted for 23.01%, 95% CI 21.30–24.72% (536/2329), and neoplastic polyps accounted for 76.99%, 95% CI 75.28–78.70% (1793/2329), 28.98% of which were both neoplastic polyps and nonneoplastic polyps (*n* = 675). According to the WHO age group, we discovered that the prevalence of neoplastic and nonneoplastic polyps was highest in the 60–74 years age group (41.22%, *n* = 950; and 10.00%, *n* = 233), and lowest in the 18–44 years age group (6.83%, *n* = 159; 4.89%, *n* = 114, respectively). A total of 1744 patients (74.88%, 95% CI 73.12–76.64%) had traditional adenoma, including 1032 patients with advanced adenoma (44.31%, 95% CI 42.29–46.33%). Overall, 59.17% of the traditional adenoma patients had advanced adenoma (95% CI 56.87–61.48% [1032/1744]). Similarly, the prevalence of both advanced and nonadvanced adenomas was also highest in the 60–74 years age group (33.26% and 20.53%, respectively) (Fig. 3).

### General characteristics of colorectal carcinoma in situ and neoplastic polyps

Our study revealed that the proportion of males among patients with CCS was 73.33% (66/90) greater than that among patients with noncarcinoma in situ 64.58% (1446/2239); however, these differences were not statistically significant (*p* = 0.088) (Table 1). The median age of patients with CCS was 66 years, which was significantly greater than the 63 years of patients with noncarcinoma in situ (*p* = 0.004). Pathology revealed that 44 patients with colorectal polyps had schistosome egg deposition in our study. Subsequent analysis revealed that patients with schistosome egg deposits had a greater risk of developing CCS (6.67% vs. 1.70%, *p* = 0.006).

Moreover, more than 4/5 (84.44%) of the CCS patients had polyps ≥ 1.0 cm in diameter, whereas the polyp diameters in noncarcinoma in situ patients were mostly < 1.0 cm (61.19%), *p* < 0.0001. Approximately 3/4 (73.29%) of patients with noncarcinoma in situ had < 3 polyps, which was greater than the 52.22% of the CCS patients (*p* < 0.0001). There were no statistically significant differences in hypertension, diabetes, coronary heart disease, MAFLD, or *Helicobacter pylori* infection between individuals with and without CCS (all *p* > 0.05).

When hematological indicators were analyzed, the findings revealed that individuals with CCS had significantly greater median CA724 levels than did those with noncarcinoma in situ (4.29U/mL vs. 2.12U/mL, *p* = 0.002). Notably, the median values of albumin and CA211 in the CCS and noncarcinoma in situ groups were the same, but the mean values of the ranks of the two groups were 972.24 vs. 1172.75 and 1446.63 vs. 1153.68, respectively, indicating that the data distributions of the two groups were different; therefore, the Mann–Whitney U test showed a significant difference (*p* < 0.05). There were no significant differences in hemoglobin, creatinine, uric acid, total cholesterol, triglyceride, CEA, AFP, or CA199 levels (*p* > 0.05).

When we analyzed the general characteristics of patients with neoplastic polyps, we found that the median age of patients with neoplastic polyps was 64 years older than that of patients with nonneoplastic polyps (59 years old, *p* < 0.0001). The proportions of multiple polyps and polyps with diameter ≥ 1.0 cm were greater in neoplastic polyps than in nonneoplastic polyps (50.81% vs. 41.42%, *p* < 0.0001; 46.35% vs. 21.27%, *p* < 0.0001, respectively). The median CA199 level of 10.97(5.88) U/mL was significantly greater than that of 9.60(6.09) U/mL in patients with nonneoplastic polyps (*p* = 0.023). Significant differences were not found in neoplastic polyps for hypertension, diabetes, MAFLD, coronary heart disease, and *Helicobacter pylori* infection (*p* > 0.05).

**Table 1:** Characteristics of patients with colorectal carcinoma in situ and neoplastic polyps

	Noncarcinoma in situ (n = 2239)	Carcinoma in situ (n = 90)	P	Nonneoplastic polyps (n = 536)	Neoplastic polyps(n = 1793)	P
Sex (male/female)	1446/793	66/24	0.088	331/205	1181/612	0.080
Age, M(IQR)	63.00(17.00)	66.00(11.00)	**0.004**	59.00(21.00)	64.00(16.00)	**< 0.0001**
Diameter of polyps	0.80(0.60)	1.50(1.50)	**< 0.0001**	0.60(0.30)	0.80(0.90)	**< 0.0001**
< 1.0 cm, n (%)	1370(61.19)	14(15.56)		422(78.73)	962(53.65)	
≥ 1.0 cm, n (%)	869(38.81)	76(84.44)		114(21.27)	831(46.35)	
Solitary polyp, n (%)	1164(52.0)	32(35.6)	**0.002**	314(58.6)	882(49.2)	**<0.0001**
Multiple polyps	1075(48.0)	58(64.4)		222(41.42)	911(50.81)	
Number of colorectal polyps			**< 0.0001**			**0.002**
< 3, n (%)	1641(73.29)	47(52.22)		416(77.6)	1272(70.9)	
≥ 3, n (%)	598(26.7)	43(47.8)		120(22.4)	521(29.1)	
Schistosome eggs	38/2201	6/84	**0.006**	8/528	36/1757	0.442
*Hp* infection	158(7.1)	5(5.6)	0.584	41(7.6)	122(6.8)	0.501
Hypertension (Yes/No)	603/1636	25/65	0.859	128/408	500/1293	0.067
Diabetes (Yes/No)	82/2157	5/85	0.385	23/513	64(1729)	0.440
CHD (Yes/No)	13/2226	0/90	1.000	3/533	10/1783	1.000
MAFLD (Yes/No)	318/1921	8/82	0.154	81/455	245/1548	0.397
WBC*10^9/L	6.05(2.09)	6.24(2.38)	0.181	6.07(2.07)	6.04(2.11)	0.845
RBC*10^12/L	4.54(0.65)	4.50(0.65)	0.267	4.54(0.66)	4.54(0.64)	0.208
Hemoglobin, g/L	138.91(20.00)	138.91(24.00)	0.617	139.00(21.00)	138.91(20.00)	0.406
PLT*10^9/L	213.00(68.00)	210.50(67.00)	0.631	213.50(67.00)	213.00(69.00)	0.902
Albumin, g/L	43.65(4.00)	43.65(2.70)	**0.005**	43.65(4.00)	43.65(5.00)	0.070
Total bilirubin, umol/L	11.90(5.50)	12.11(3.18)	0.678	11.65(5.70)	12.10(5.20)	0.522
ALT, U/L	19.00(14.00)	23.84(11.00)	0.412	20.00(15.00)	19.00(13.00)	0.412
AST, U/L	20.00(8.00)	21.50(5.00)	0.302	19.00(8.00)	20.00(7.00)	0.302
γ-GT, U/L	25.00(18.10)	34.09(17.10)	0.258	24.00(19.10)	26.00(18.10)	0.070
ALP, U/L	73.00(20.00)	73.05(11.00)	0.051	73.00(22.00)	73.05(20.00)	0.843
LDH, U/L	160.08(32.00)	160.08(13.60)	0.866	160.08(33.80)	160.08(31.00)	0.808
Creatinine, umol/L	74.60(20.00)	74.60(13.00)	0.345	74.00(21.00)	74.60(19.00)	0.494
Uric acid, umol/L	330.48(98.00)	330.48(48.50)	0.221	330.48(110.00)	330.48(89.00)	0.512
TC, mmol/L	4.51(0.97)	4.51(0.51)	0.103	4.51(0.92)	4.51(0.96)	0.488
TG, mmol/L	1.60(0.72)	1.68(0.48)	0.157	1.58(0.75)	1.62(0.71)	0.844
AFP, ng/mL	3.01(1.26)	3.01(1.12)	0.288	2.88(1.42)	3.01(1.21)	0.315
CEA, ng/mL	2.48(1.28)	2.57(0.53)	0.450	2.34(1.30)	2.56(1.26)	0.146
CA125, U/mL	10.17(3.77)	10.52(2.36)	0.240	9.75(4.28)	10.34(3.60)	0.184
CA199, U/mL	10.44(6.01)	11.25(3.42)	0.055	9.60(6.09)	10.97(5.88)	**0.023**
CA724, U/mL	2.12(3.18)	4.29(2.77)	**0.002**	2.05(3.22)	2.20(3.16)	0.457
CA211, ng/mL	2.61(1.05)	2.61(0.79)	**< 0.0001**	2.56(1.11)	2.61(1.05)	0.287

*Hp*, *Helicobacter pylori*; CHD, coronary heart disease; MAFLD, metabolic associated fatty liver disease; WBC, white blood cell; RBC, red blood cell; PLT, platelet; ALT, alanine aminotransferase; AST, aspartate aminotransferase; γ-GT, γ-glutamyl transpeptidase; ALP, alkaline phosphatase; LDH, lactate dehydrogenase; TC, total cholesterol; TG, triglycerides; CEA, carcinoembryonic antigen; AFP, alpha fetoprotein; CA, carbohydrate antigen. Data with significant differences of p < 0.05 were shown in bold

Data with significant differences of p < 0.05 were shown in bold

### Potential risk factors for colorectal carcinoma in situ

Variables that were significantly different in the bivariate analysis were included in the univariate logistic regression analysis. The results of univariate logistic regression analysis were shown in Table 2. The results showed that age, diameter ≥ 1.0 cm, number of polyps ≥ 3, schistosome egg deposition, CA724, and CA211 level were risk factors for colorectal carcinoma in situ, while the serum albumin concentration was a protective factor.

Then, based on the findings of the univariate logistic regression analysis, variables with *p* < 0.05 were included in the multivariate logistic regression analysis, and the Hosmer and Lemeshow test was performed to assess goodness of fit. A diameter ≥ 1.0 cm, number of polyps ≥ 3, schistosome egg deposition, CA724, and CA211 were risk factors for CCS (Table 2). The risk of CCS in patients with a colorectal polyp diameter ≥ 1.0 cm was 8.07 times greater than that in patients with a diameter < 1.0 cm (OR 8.07, 95% CI 4.48–14.55; *p* < 0.0001). Moreover, the risk of CCS in patients with polyps ≥ 3 was 1.98 times greater than that in patients with polyps < 3 (OR 1.98,95% CI 1.27–3.09; *p* = 0.002). Patients with schistosome egg deposits also had a significantly increased risk of CCS (OR 2.70,95% CI 1.05–6.98), *p* = 0.040. In addition, the higher the levels of CA724 (OR 1.01,95% CI 1.00–1.02) and CA211 (OR 1.16,95% CI 1.03–1.32) in patients with colorectal polyps were, the greater the risk of CCS. The Hosmer and Lemeshow test indicated that the goodness of fit was good (χ^2^ = 2.87, *p* = 0.942).

**Table 2:** Univariate and multivariate logistic regression analyses of colorectal carcinoma in situ

Variables	Univariate logistic regression			Multivariate logistic regression	
	OR (95% CI)	P		OR (95% CI)	P
Age	1.03 (1.01–1.05)	0.004		1.01 (0.99–1.03)	0.277
Diameter of polyps (< 1.0 cm, ref.)					
≥ 1.0 cm	8.56 (4.81–15.23)	< 0.0001		8.07 (4.48–14.55)	< 0.0001
Number of colorectal polyps (< 3, ref.)					
≥ 3	2.51 (1.64–3.84)	< 0.0001		1.98 (1.27–3.09)	0.002
Schistosome egg deposition (No, ref.)					
Yes	4.14 (1.7–10.06)	0.002		2.70 (1.05–6.98)	0.040
Albumin	0.92 (0.87–0.97)	0.004		0.96 (0.91–1.02)	0.215
CA724	1.01 (1.00–1.02)	0.001		1.01 (1.00–1.02)	0.012
CA211	1.18 (1.06–1.30)	0.002		1.16 (1.03–1.32)	0.018

CA724, carbohydrate antigen 724; CA211, carbohydrate antigen 211

### Potential risk factors for colorectal neoplastic polyps

**Fig. 4:**
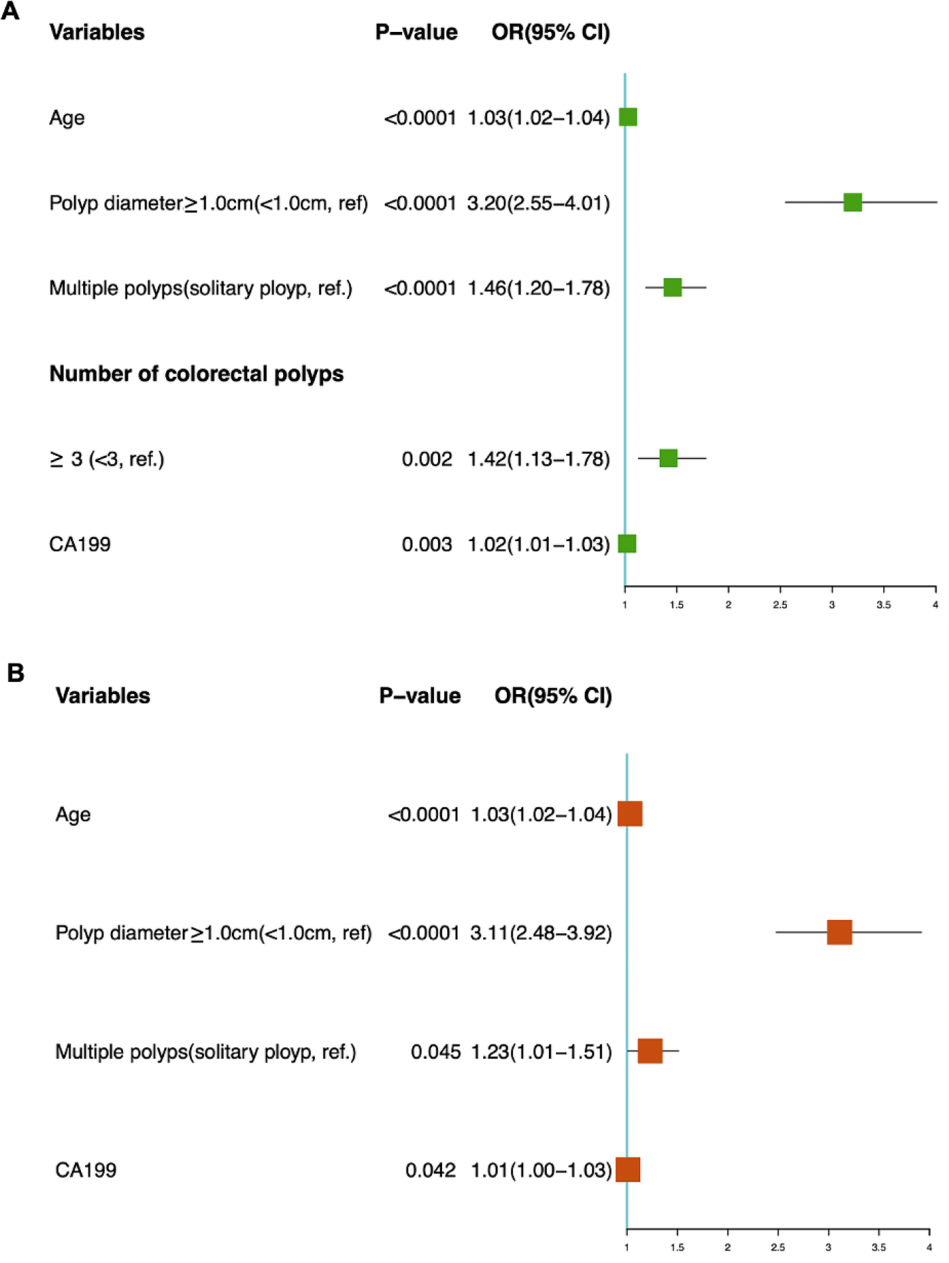
Logistic regression analysis of colorectal neoplastic polyps. (**A**) Univariate logistic regression; (**B**) Multivariate logistic regression. CA199, carbohydrate antigen 199

Age (OR 1.03, 95% CI 1.02–1.04), polyp diameter ≥ 1.0 cm (OR 3.20, 95% CI 2.33–4.01), multiple polyps (OR 1.46, 95% CI 1.20–1.78), number of polyps ≥ 3 (OR 1.42, 95% CI 1.13–1.78), CA199 (OR 1.02, 95% CI 1.01–1.03) were risk factors for neoplastic polyps when analyzed patients with colorectal neoplastic or nonneoplastic polyps (Fig. 4A).

When multivariate logistic regression analysis was carried out, multiple polyps and polyps ≥ 3 were considered to be similar. Multiple polyps were selected in the analysis, and the Hosmer and Lemeshow test suggested that the goodness of fit was good (χ^2^ = 7.48, *p* = 0.486). This study showed that for each 1-year increase in age, the risk of neoplastic polyps increased by 3% (OR 1.03, 95% CI 1.02–1.04), *p* < 0.0001. Compared with patients with a colorectal polyp diameter < 1.0 cm, patients with a polyp diameter ≥ 1.0 cm had a 2.11-fold greater risk of neoplastic polyps (OR 3.11, 95% CI 2.48–3.92), *p* < 0.0001. In addition, multiple polyps and CA199 levels were also risk factors for the occurrence of neoplastic polyps (Fig. 4B).

## Discussion

This study revealed the prevalence of diverse pathological colorectal polyps. Of these, 3.86% of patients had CCS. This study observed that tubular adenoma and tubulovillous adenoma accounted for a large proportion of the population or all the detected polyps. The presence of tubular, villous, or serrated structures affects the risk of carcinogenesis of these lesions. In addition, the appearance of villous structures, accompanied by a high degree of cellular dysplasia and a larger polyp diameter, increases the malignant potential of adenomas [12].

A recent study indicated that the 10-year cumulative incidence of colorectal cancer in patients with hyperplastic polyps was 1.6%, while the cumulative incidence in the general population was 2.1% [8], indicating that hyperplastic polyps do not increase the risk of colorectal cancer. Prospective studies have also shown that the presence of hyperplastic polyps does not increase the risk of adenoma recurrence [14]. Participants with conventional adenomas were more likely to develop colorectal cancer than were those without polyps, with a hazard ratio (HR) of 2.61 (95% CI 1.93–3.52), and this positive correlation was driven by advanced adenomas (HR 4.07, 95% CI 2.89–5.72; *p* < 0.001). The 5-year and 10-year cumulative incidences of colorectal cancer in participants without polyps were 0.2% and 0.4%, compared with 0.1% and 0.3%, respectively, for nonadvanced adenomas and 0.6% and 1.7%, respectively for advanced adenomas [13]. We know that neoplastic polyps have a certain risk of becoming malignant, while nonneoplastic polyps have almost no risk. Identifying the risk factors for neoplastic polyps is crucial because these patients are at risk of becoming cancerous.

Therefore, to identify high-risk individuals as soon as possible, we further investigated the risk factors for CCS and neoplastic polyps. We found that ≥ 3 colorectal polyps and a diameter ≥ 1.0 cm were risk factors for CCS. HE et al. [13] also revealed that large size (≥ 10 mm) and multifocality (3–10 tubular adenomas) of tubular adenomas were related to an increased risk of colorectal cancer, with HRs of 3.40 (95% CI 1.86–6.24, *p* = 0.001) and 3.15 (95% CI 1.29–7.67, *p* = 0.01), respectively. These findings correspond to our findings.

Schistosomiasis is a tropical parasitic disease. After human infection with schistosomiasis, eggs are excreted through the feces, which can cause intestinal schistosomiasis and other diseases [15]. The presence of schistosome egg deposits in the colon indicated previous schistosome infection, and schistosome egg deposits, according to our findings, were a risk factor for CCS. We hypothesized that patients who have previously been infected with schistosomes may be more susceptible to developing CCS or even colorectal cancer.

Some studies have shown that individual lifestyle, age, and Western dietary patterns are associated with the risk of colorectal adenoma, smoking is associated with the occurrence of serrated polyps, diabetes is associated with sessile serrated lesions, and a family history of colorectal cancer is not associated with adenoma or serrated polyps [16, 17]. No significant differences in sex, hypertension, diabetes status, coronary heart disease status, MAFLD status, or *Helicobacter pylori* infection status were found in patients with CCS or with neoplastic polyps in this investigation. However, we observed that the prevalence of neoplastic polyps or advanced adenoma was the highest in the 60–74 years age group. We suspect that colonoscopy examination for this age group may require extra attention.

Moreover, our findings suggested that tumor marker levels may have changed slightly in individuals with CCS or neoplastic polyps. The CEA, which was first identified in 1965, is generated by endodermal epithelial tumor cells in the digestive tract [18]. It is a tumor marker used to diagnose colorectal cancer but is rarely utilized for detection in its early stages [19]. Neither neoplastic polyps nor CCS were affected by CEA. We think that since CCS was an early cancer lesion, in vivo CEA molecular markers may not have changed. Although the serum CEA is a reliable indicator of colorectal cancer, according to the European Group on Tumor Markers guidelines, 20–30% of patients still have nonelevated CEA levels even in patients with advanced tumors. Patients without elevated CEA levels may benefit from additional tumor markers, such as CA199, CA242, CA724, and CA50. Like CEA, CA199 and CA242 cannot be utilized to identify colorectal cancer in its early stages [20]. CA724 is associated with colorectal cancer and is considered to be a crucial early marker of colorectal cancer and/or other dysplastic colonic disorders [21]. Our research demonstrated that CA724 (OR 1.01, 95% CI 1.00–1.02) and CA211 (OR 1.16, 95% CI 1.03–1.32) levels were associated with CCS, also known as high-grade intraepithelial neoplasia, severe dysplasia, or very early colorectal cancer. Perhaps the CA724 and CA211 could help with the early detection of colorectal cancer.

Large sample sizes and extensive data, including blood cell, biochemical marker, blood lipid, tumor marker, and other indicator data, were used in our investigation. Additionally, the data sources used were objective values, which helped guarantee the legitimacy and veracity of the study.

Certainly, this study has several limitations. First, we were unable to track changes in the prevalence of colorectal cancer over time because this study was retrospective. Second, 1133 participants (48.5%) in this study had more than one colorectal polyp. Due to the high prevalence of multiple colorectal polyps and the complex statistics of polyp locations, the locations of CCS and neoplastic polyps were not investigated or analyzed in this study. Furthermore, we did not examine the impact of body mass index on colorectal polyps, but differences in MAFLD, hypercholesterolemia, or hypertriglyceridemia were not detected in either CCS or neoplastic polyps. Finally, the participants of the study were from a single center, and whether the findings can be widely applied needs to be verified by further multicenter studies.

The present study investigated the prevalence of colorectal polyps of diverse pathological types and the risk factors for CCS and neoplastic polyps. Our research may have clinical practical implications for early detection of high-risk polyps and the population at high risk of colorectal cancer, as well as for enhancing the efficiency of colonoscopy.

## Data Availability

Not Applicable.
